# Putting piezoelectric sensors into Fano resonances

**DOI:** 10.1038/s41378-024-00847-6

**Published:** 2024-12-24

**Authors:** Mengting Wang, Jianqiu Huang, Qing-An Huang

**Affiliations:** Key Laboratory of MEMS of the Ministry of Education, Southeast University, Nanjing, 210096 China

**Keywords:** Electrical and electronic engineering, Organic-inorganic nanostructures

## Abstract

Piezoelectric resonance sensors are essential to many diverse applications associated with chemical and biological sensing. In general, they rely on continuously detecting the resonant frequency shift of piezoelectric resonators due to analytes accreting on their surfaces in vacuum, gas or fluid. Resolving the small analyte changes requires the resonators with a high quality factor. Here, we propose theoretically and demonstrate experimentally a scheme using a physics concept, i.e., a Fano resonance, to enhance the quality factor rather than optimizing the structure and material of the resonator itself though these are important. The Fano resonance arises due to the interference between a discrete mode and a continuum of modes, leading to the asymmetric and steep dispersion. In our scheme, the as-fabricated piezoelectric sensors are put into the Fano resonance by connecting an external shunt capacitor to them. As a verification case, one-port surface acoustic wave (SAW) resonators on LiNbO_3_ substrate, incorporating a composite of polymethyl methacrylate (PMMA) and graphene oxide (GO) for humidity sensing, have been fabricated and characterized. We enhance the quality factor by up to a factor of about 8, from 929 for the as-fabricated sensor to 7682 for that with the external shunt capacitor. Our results pave the way for the practical development of piezoelectric resonance sensors with high quality factor.

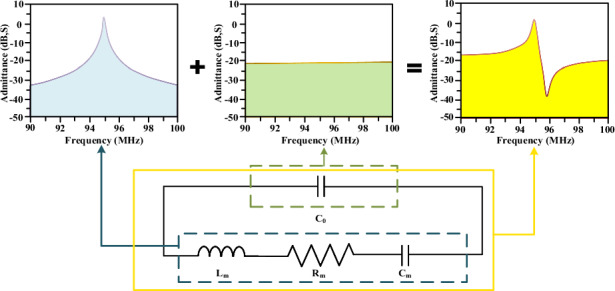

## Introduction

A piezoelectric resonance sensor has a long history of research dating back to 1959^1^, and Sauerbrey first recognized that the resonance frequency of thickness-shear mode (TSM) quartz resonators alters linearly when a foreign mass is deposited on the quartz surface in air or vacuum. From the resonant frequency shift, it was possible to detect mass deposition on the quartz surface with a rather extreme sensitivity. Since then, by incorporating various sensing materials and utilizing different vibration modes of the resonator^2–10^, the piezoelectric resonance sensor has found a wide range of applications for chemical^11–14^ and biological sensing^15–20^, as well as in situ interface interaction research^21–24^. In order to resolve small analyte variations, the resonators are required to have a high quality factor, especially when they are operated in vapor or liquid environments. Traditionally, approaches to enhance the quality factor are to optimize the vibration modes^17,25^, structure^26^, and materials^27,28^ of the resonators. In this article, we propose another method to enhance the quality factor of the as-fabricated piezoelectric resonance sensors by utilizing Fano resonances. The proposed method can be easily realized by only connecting an external shunt capacitor to the piezoelectric resonance sensor.

The Fano resonance is a ubiquitous scattering wave phenomenon^29,30^. It occurs when a discrete quantum state interferes with a continuum band of states. While originally found in atomic and solid states physics, the ultrasharp spectrum of the Fano resonance promises applications for a broad range of photonic devices^31,32^. Moreover, by exciting the Fano resonance effect in photonic sensors near the frequency of interest through the design optimization and fabrication control, the narrow linewidth of the Fano resonances leads to high resolution sensing due to the high quality factor^33^. On the other hand, an analogy of the Fano resonances to classical resonances in a harmonic electrical oscillator system can also be realized^34,35^. Inspired by these works, here we explore the consequence of as-fabricated piezoelectric resonance sensors for enhancing the quality factor using the Fano resonances.

## Operation principle

The asymmetry of the Fano resonance can be characterized by^30^

1 $$\sigma =\frac{{(\varepsilon +q)}^{2}}{{\varepsilon }^{2}+1}\times H+{\sigma }_{0}$$

where *q*, $\varepsilon$, *H*, and ${\sigma }_{0}$ represent the phenomenological shape parameter, the reduced energy, gain parameters, and the offset, respectively. For resonators characterized by the impedance spectrum, the Q factor of a symmetric Lorentz resonance as well as an asymmetric Fano resonance can be fitted by^36^

2 $$Q=\frac{{f}_{s}}{2g\sqrt{\sqrt{2}-1}}\left(1-\sqrt{\left|q\right|}\right)+\frac{{f}_{s}^{2}+{g}^{2}}{2g{f}_{s}}\sqrt{\left|q\right|}$$

where *g* is the linewidth and ${f}_{s}$ is the series resonance frequency.

**Fig. 1:**
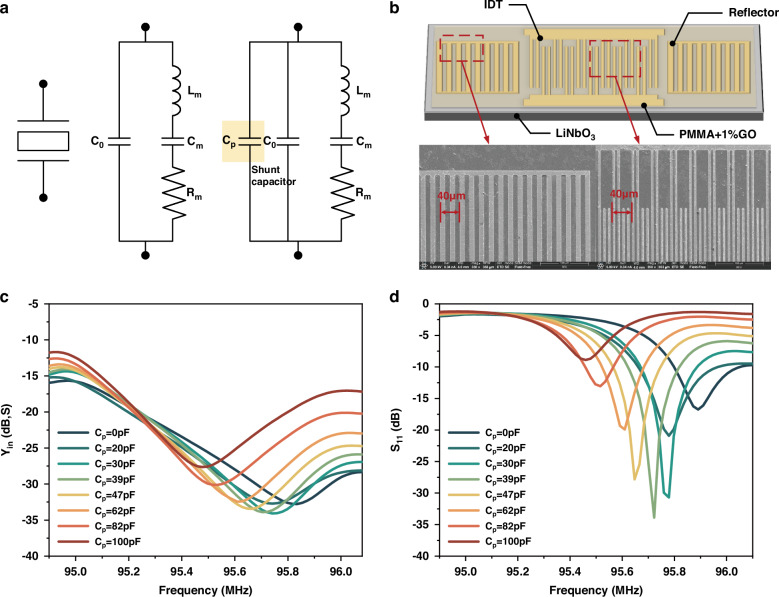
Piezoelectric resonance sensors operating at Fano-like resonances. **a** The equivalent circuit representation of the piezoelectric resonator, and an external shunt capacitor is connected to the resonator. **b** Schematic diagram and partial SEM image of the fabricated SAW resonator for humidity sensing. **c** The measured admittance (Y_11_) as a function of frequency for different shunt capacitances. **d** The measured scattering parameter (S_11_) as a function of frequency for different shunt capacitances. *C*_*p*_ = *0* in (**c**, **d**) corresponds to the as-fabricated resonator

To describe how the quality factor of as-fabricated piezoelectric resonance sensors is enhanced by utilizing Fano resonances, we present an analysis based on the Butterworth-van Dyke (BVD) equivalent circuit for the piezoelectric resonator^10^. As shown in Fig. 1a, the equivalent circuit consists of a static capacitance (*C*_*0*_) in parallel with a motional branch (*L*_*m*_*, C*_*m*_*, R*_*m*_). *C*_*0*_ accounts for the electrode capacitance of the resonator, *R*_*m*_ is the motional resistance representing damping, *L*_*m*_ is the motional inductance representing the vibrating mass, and *C*_*m*_ is the motional capacitance representing the stored energy. The motional branch of the resonator is due to contributions from the electromechanical or piezoelectric characteristics of the resonator. By measuring the electrical response of the unloaded resonator over a range of frequencies near resonance and fitting the equivalent circuit model to these data, the values for *L*_*m*_*, C*_*m*_*, R*_*m*_, and *C*_*0*_ can be extracted (See Method).

For an unloaded piezoelectric resonator, the admittance is given by

3 $${Y}_{{in}}=i\omega {C}_{0}+\frac{1}{\frac{1}{i\omega {C}_{m}}+i\omega {L}_{m}+{R}_{m}}$$

where $\omega =2\pi f$ is the angular frequency. As seen from the circuit in Fig. 1a, it approximately yields two fundamental resonance frequencies at the maximum and minimum of the admittance amplitude, respectively,

4 $${f}_{s}\approx \frac{1}{2\pi \sqrt{{L}_{m}{C}_{m}}}$$

5 $${f}_{p}\approx \frac{1}{2\pi \sqrt{{L}_{m}\frac{{C}_{m}{C}_{0}}{{C}_{m}+{C}_{0}}}}$$

The motional inductance *L*_*m*_ and capacitance *C*_*m*_ produce a ${f}_{s}$, the static capacitance *C*_*0*_ and the elements in the motional branch produce a parallel resonant frequency (${f}_{p}$), and *R*_*m*_ accounting for the loss determines the linewidth of the resonance. For the piezoelectric resonance sensors, analytes accreting on the surface of the resonator cause the vibrating mass to change, leading to the resonance frequency shift. The shift in resonance frequency due to mass loading effects can be observed by impedance or S-parameter spectrum. They are equivalent in measuring the series resonant frequency shift^37,38^.

Essentially, the admittance of the series motional branch exhibits the Lorentz spectrum, and the admittance of the parallel static capacitance branch may be considered to be the continuous spectrum (See supplementary). As a result, the Fano-like resonance occurs for the piezoelectric resonator. In fact, however, the static capacitance of the practical piezoelectric resonance sensors is too small for the Fano resonance to be clearly observed. In the extreme case where ${C}_{0}=0$, the admittance of the piezoelectric resonator reduces to the series motional branch exhibiting only the Lorentz spectrum. This is the reason why the circuit parameter extraction of piezoelectric resonance sensors is usually operated according to the Lorentz spectrum^39^. On the other hand, if the static capacitance was made large enough (the extreme case: ${C}_{0}\to \infty$), the system would approximately approach a short circuit, giving rise to a significant decrease in amplitude, and the Fano resonance could not clearly be observed either. In order to make the piezoelectric resonant sensor exhibit the Farno resonance, we connect an external capacitor *C*_*p*_ in parallel to the static capacitor *C*_*0*_. For the given *R*_*m*_, *C*_*p*_ is adjusted in such a way that the piezoelectric resonant sensor is put into the Fano resonance, leading to the high quality factor.

To demonstrate the scheme, the scattering parameter ${S}_{11}$ is utilized since the quality factor of the piezoelectric resonant sensor does not depend on what measurement methods (admittance, impedance, or S-parameters) are used^40^.

The scattering parameter ${S}_{11}$ reads

6 $${S}_{11}=\frac{{Y}_{0}-{Y}_{{in}}}{{Y}_{0}+{Y}_{{in}}}$$

where ${Y}_{0}=1/{Z}_{0}$ and$\,{Z}_{0}$ is the characteristic impedance of the signal source (usually ${Z}_{0}=50$ Ω). Taking $\partial \left|{S}_{11}\right|/\partial f=0$, it yields the frequency (*f*_*min*_) at which ${S}_{11}$ gets the minimum ${\left|{S}_{11}\right|}_{\min }$ (See supplementary)

7 $${f}_{\min }\approx \left[1+\frac{{C}_{m}}{2\left({C}_{0}+{C}_{p}\right)}\right]{f}_{s}.\,$$

It shows that ${f}_{\min }$ is situated between ${f}_{s}$ and ${f}_{p}$, and ${f}_{\min }$ is proportional to ${f}_{s}$. For impedance spectrum, the Q factor can be characterized by Eq. (2)^36^. For the S_11_ spectrum, the Q factor is given by^41^

8 $$Q\approx \frac{{f}_{\min }}{\Delta {f}_{3{dB}}}$$

where $\Delta {f}_{3{dB}}={f}_{2}-{f}_{1}$ is the frequency bandwidth, and ${f}_{1}$ and ${f}_{2}$ are the frequencies corresponding to a response value of $\left|{S}_{11}\right|/{\left|{S}_{11}\right|}_{\min }=\sqrt{2}$. The scattering parameter ${S}_{11}$ is measured in different external shunt capacitance ${C}_{p}$, and Q is then estimated by Eq. (8).

## Experiments and results

**Fig. 2:**
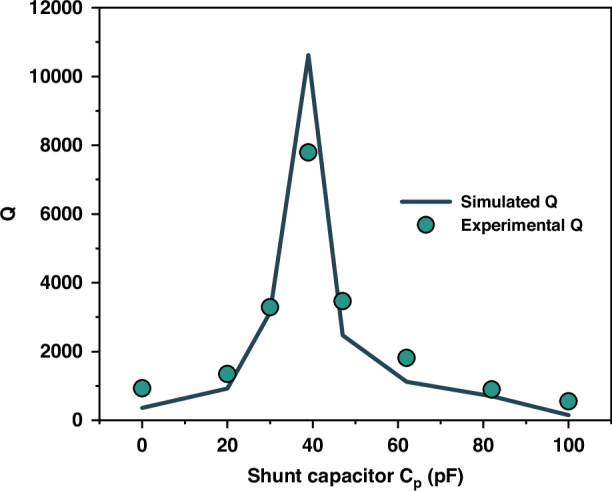
The measured and simulated quality factor as a function of shunt capacitance. Extracted parameters of circuit elements for the as-fabricated resonator are used for simulations. They are ${C}_{0}=96.16\,{\rm{pF}}$, ${C}_{m}=1.737\,{\rm{pF}}$, ${L}_{m}=1.616\,{\mu }{\rm{H}}$, and ${R}_{m}=2.994\,{\Omega }$, respectively

We demonstrate the analysis presented above in one-port SAW resonators on LiNbO_3_ substrate incorporating a composite of PMMA and GO for humidity sensing (See Method), as shown in Fig. 1b. Previously, piezoelectric resonators with a shunt capacitor have been used to diminish noise and vibration in mechanical structures^42^ or to compensate for non-ideal phase-frequency characteristic of the active components in oscillator circuits^40^. Our work differs in that we aim to demonstrate a high quality factor scheme. Figures 1c, d show the measured admittance $\left|{Y}_{{in}}\right|$ and scattering parameter $\left|{S}_{11}\right|$ as a function of frequency at different shunt capacitances. It is evident from the admittance spectrum that the Fano-like resonance is not obvious for the as-fabricated resonator (${C}_{p}=0$). To enhance the quality factor, 3 dB bandwidth is desired to be narrow, that is, the series resonant frequency *(f*_*s*_) and parallel resonant frequency (*f*_*p*_) are adjusted as close as possible by regulating *C*_*p*_. However, as indicated in Eqs. (4), (5) and (7), *f*_*min*_ is situated between *f*_*s*_ and *f*_*p*_. Both *f*_*p*_ and *f*_*min*_ decrease with the increase of the shunt capacitance, while *f*_*s*_ decreases slightly as the shunt capacitance increases. As a result, there exists an optimization *C*_*p*_ by which the quality factor gets its maximum. Figure 2 shows the estimated quality factor as a function of the shunt capacitance. It equals 929 for the as-fabricated resonator (${C}_{p}=0$), and 7682 for the optimization shunt capacitance (${C}_{p}=39\,{\rm{pF}}$). Attention should be drawn to the fact that in some applications the piezoelectric element may be bridged at the input and output terminals of the feedback circuit^43,44^, with the shunt capacitance representing a parasitic feedthrough effect. The line shape of the Fano resonance can also be observed in the equivalent circuit of a phase-locked loop. There are schemes that provide adaptive subtraction of the crosstalk effect copy modulating the phase response, and schemes that isolate the feedback effect by providing sufficient electromechanical coupling^43,44^. The proposed shunt technique can also be considered for future applications to phase-locked loop modulation.

We perform simulations using ADS (Advanced Design System) software (See Supplementary). Parameters for the simulations are extracted from the as-fabricated resonator for humidity sensing. They are, respectively,$\,{C}_{0}=96.16\,{\rm{pF}}$, ${C}_{m}=1.737\,{\rm{pF}}$, ${L}_{m}=1.616\,{\rm{\mu }}{\rm{H}}$, and ${R}_{m}=2.994\,\Omega$. The admittance and scattering parameter have been simulated for different shunt capacitances, and the quality factor is estimated according to Eq. (8). Figure 2 shows the simulated quality factor as a function of the shunt capacitance. The results of the ADS simulation are generally in good agreement with the corresponding results obtained experimentally.

**Fig. 3:**
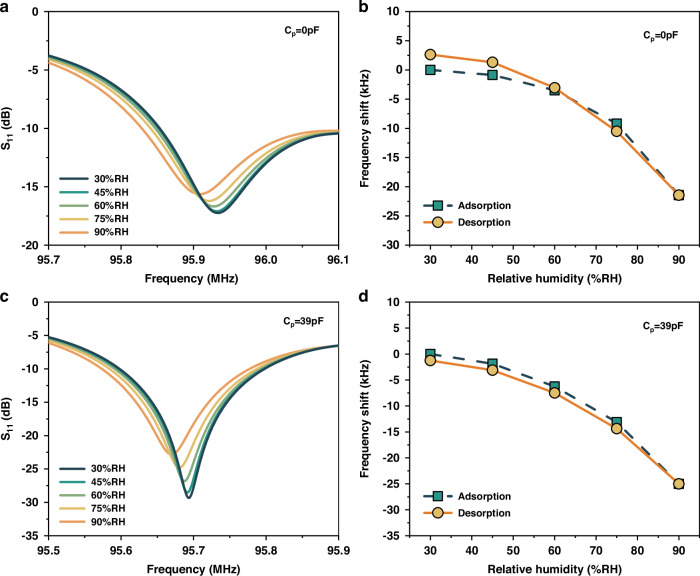
The humidity response of the SAW resonator. **a**, **c** The measured scattering parameter (S_11_) as a function of frequency at different humidity levels for *C*_*p*_ = *0* and *C*_*p*_ = *39* *pF*, respectively. **b**, **d** The measured resonant frequency shift as a function of relative humidity for *C*_*p*_ = *0* and *C*_*p*_ = *39* *pF*, respectively. *C*_*p*_ = *0* in (**a**, **b**) corresponds to the as-fabricated resonator. Adsorption: the humidity level increases from 30% RH to 90% RH. Desorption: the humidity level decreases from 90% RH to 30% RH

On the basis of the simulation, we fabricated a prototype of a one-port SAW resonator on a 128° Y-X LiNbO_3_ substrate exhibiting shear horizontal mode (SHM). As shown in Fig. 1b, a composite of PMMA and GO was utilized here which indicates the good static, dynamic, and repeatable properties for humidity sensing^45^. Humidity sensors have found wide applications in industry, agriculture, and daily life^46^. Nevertheless, they are tremendously desired with high resolution for medical fields in respiration monitoring, infusion pumps, ventilators, diagnostic instruments, and medicine storage^47^. We measured the humidity response of the SAW resonator from 30% RH to 90% RH for different shunt capacitances (See Method). Figure 3a shows the measured scattering parameter ${S}_{11}$ as a function of frequency for the as-fabricated SAW resonator (${C}_{p}=0$). As shown in Fig. 3b, the resonant frequency shift as a function of humidity level is obtained according to Fig. 3a. Figure 3c, d show the corresponding results by using the optimization shunt capacitance (${C}_{p}=39\,{\rm{pF}}$). The composite of PMMA and GO on the resonator is sensitive to ambient humidity. It adsorbs water molecules from the surrounding, leading to the mass increase and in turn a decrease in resonant frequency of the resonator. The sensitivity of the humidity sensor is defined as the resonant frequency shift (*Δf*) in response to relative humidity variations (*ΔRH*), i.e., $S=\Delta f/\Delta {RH}$. From Fig. 3b, d, the sensitivity is estimated to be approximately 0.358 kHz/% RH. There is almost no change in sensitivity before and after the shunt capacitor is connected. However, the quality factor using the optimization shunt capacitance is greatly increased. To evaluate the overall performance of the sensor, the figure of merit (FOM) for the Fano resonance in optical sensing is adopted here^32^. The FOM is defined as the sensitivity S divided by the full width at half maximum, i.e., ${FOM}=S/\Delta {f}_{3{dB}}$. Then, the FOM of our sensor is significantly increased from 0.006 (%RH)^−1^ for ${C}_{p}=0$ to 0.044 (%RH)^−1^ for ${C}_{p}=39\,{\rm{pF}}$.

Table 1 provides a comparison of the performance of various types of resonant humidity sensors^48–52^, indicating that our sensor performs well in terms of the quality factor.

**Table 1:** Comparison of different humidity sensors

Sensing material	Resonance frequency	Range (%RH)	Response value	Q factor	Type	Ref.
ZnO	1400 MHz	20–50	-3.4 kHz/%RH	530	FBAR	^48^
PI	1055 MHz	15–85	67.3 kHz/%RH	210	FBAR	^49^
polymer	130 MHz	40–85	~155.6 Hz/%RH	706	SAW	^50^
ZnO NRs (1.5${\rm{\mu }}{\rm{m}}$)	~16.5KHz	30–80	~0.72 Hz/%RH	~2000	Piezoresistive	^51^
GO/PMMA	95.72 MHz	30–90	358 Hz/%RH	7682	SAW	this work

## Conclusions

In summary, we have demonstrated experimentally that the quality factor of as-fabricated piezoelectric sensors can greatly be increased by putting them into Fano resonances via using an external shunt capacitance. The quality factor enhancement has been validated on the SAW resonator for humidity sensing. Furthermore, as long as piezoelectric resonant devices can be represented by the BVD model, their quality factor could be adjusted by the shunt capacitance. Consequently, our scheme can, in principle, be applied in other types of piezoelectric resonant sensors such as quartz crystal microbalance (QCM) and film bulk acoustic wave resonators (FBAR). However, at higher frequencies, an external shunt capacitor connected to the piezoelectric resonance sensor would bring parasitic effects such as parasitic resistance or inductance, which needs to be studied, for instance, the external shunt capacitance could also be integrated on-chip with the resonators. Our work opens up an avenue to resolving the small analyte changes for chemical and biological sensors.

## Methods

### Design and fabrication of SAW resonators for humidity sensing

A SAW resonator may be visualized as a crystal resonator in which the acoustic energy is confined entirely to one surface of the substrate^53^. As shown in Fig. 1b, a one-port SAW resonator incorporating humidity sensing films has been designed and fabricated on a 128° Y-X LiNbO_3_ substrate with a thickness of 400 μm. A 200 nm gold (Au)/ 15 nm chromium (Cr) layer was used to form the interdigital transducer (IDT) in the central area with Bragg mirrors placed on both sides. The distributed reflectors consist of 200 reflecting Au stripes, each with a finger width of 10 μm and a period of 20 μm. The IDT consists of 40 finger pairs, each with a finger width of 5 μm.

The period of the IDT is determined by the following equation^54^

9 $$p=\frac{{\lambda }_{0}}{2{f}_{0}}=\frac{{\upsilon }_{s}}{2{f}_{0}}$$

where ${\lambda }_{0}$ is the wavelength of the SAW resonator; ${\upsilon }_{s}$ is the velocity of surface acoustic waves; and ${f}_{0}$ is the fundamental resonant frequency. When the finger width is set to an integer multiple of the half-wavelength of the SAW resonator, the acoustic waves are superimposed in phase, resulting in maximum excitation intensity. Therefore, the split finger width is set to 5 μm, resulting in a resonant frequency of approximately 96 MHz.

An Au film with a Cr adhesion layer was deposited onto the LiNbO_3_ substrate by an electron beam evaporation process. Then it was patterned and etched to form the SAW structure. Finally, the sensitive material was electrospun on the structure from a DMF (N,N-Dimethylformamide, ACS Spectral grade, ≥99.8%) solution of 20% wt PMMA and 1% wt GO^45^.

### Extraction of circuit parameters of SAW resonators

As shown in Fig. 1a, a SAW resonator is represented by equivalent circuit parameters. The admittance as a function of frequency is first measured for the as-fabricated SAW resonator. The series resonant frequency (${f}_{{\rm{s}}}$) and parallel resonant frequency (${f}_{{\rm{p}}}$) can be obtained by the maximum and minimum of the measured admittance. From Eq. (3), it is apparent that at an ultra-high frequency (UHF) the admittance is approximately equal to $i\omega {C}_{0}$. Thus, ${C}_{0}$ can be extracted by the slope of the admittance spectrum in the UHF range. According to Eqs. (4) and (5), ${C}_{m}$ can approximately be obtained by^10^

10 $${C}_{m}={C}_{0}\left[{\left(\frac{{f}_{p}}{{f}_{s}}\right)}^{2}-1\right]$$

${L}_{m}$ and ${R}_{m}$ can be extracted by, respectively,

11 $${L}_{m}=\frac{1}{4{\pi }^{2}{f}_{s}^{2}{C}_{m}}$$

12 $${R}_{m}=\frac{1}{Q{\omega }_{s}{C}_{m}}$$

where Q is the quality factor of the unloaded SAW resonator.

### Measurements of SAW resonators for humidity sensing

A test system is shown (See Supplementary Fig. S7). The SAW sensor was placed inside a sealed container, which was connected to a humidity generator (Zonho-tech STA-EMG). The humidity generator adjusted humidity levels by varying the ratio of injected water and nitrogen, and a split temperature and humidity transmitter (Race M&C-Tech HTDT2-IESX102) were used for calibration. A vector network analyzer VNA (Copper Mountain PLANAR TR1300/1) was utilized to characterize the output of the sensor. For each shunt capacitor, the test frequency was swept from 94.6 to 96.1 MHz with 1600 sample points. Both adsorption and desorption processes for humidity sensing were measured.

## Supplementary information

Supplementary-Putting piezoelectric sensors into Fano resonances

## Data Availability

The data that support the plots within this paper and other findings of this study are available from the corresponding author upon reasonable request.
