# Supplementary material for: Putting piezoelectric sensors into Fano resonances
Source: Microsyst Nanoeng. 2024 Dec 24;10:202. doi: 10.1038/s41378-024-00847-6 (PMC11668863; doi:10.1038/s41378-024-00847-6)
Supplement: Supplementary file 1 — Supplementary-Putting piezoelectric sensors into Fano resonances [file 41378_2024_847_MOESM1_ESM.pdf]

SUPPLEMENTARY INFORMATION

Putting piezoelectric sensors into Fano resonances

Mengting Wang[1], Jianqiu Huang[1*] & Qing-An Huang[1*]

1 Key Laboratory of MEMS of the Ministry of Education, Southeast University, Nanjing 210096, China.
*e-mail: hjq@seu.edu.cn; hqa@seu.edu.cn

A. The admittance, scattering parameter, and quality factor of piezoelectric resonance sensors

B. Simulation of the admittance, scattering parameter, and quality factor of piezoelectric resonance sensors

C. Fabrication process of piezoelectric resonance sensors

D. Experimental set-up

A. The admittance, scattering parameter, and quality factor of piezoelectric resonance sensors

A.1. An analogy of Fano resonances to piezoelectric resonance sensors

The Fano resonance is a ubiquitous scattering wave phenomenon. It occurs when a discrete quantum state interferes with a continuum band of states. As shown in Fig. S1, in the BVD model, the admittance of the L_m, C_m, and R_m series branch (the acoustic branch) displays a typical symmetrical Lorentz shape, while the admittance of the parallel C_0 branch (the electric branch) is equivalent to a continuous flat background. The superposition of the two branches can produce the phenomenon of Fano-like resonance, implying that SAW resonators inherently possess the Fano resonance characteristics. In fact, however, the static capacitance C_0 of the practical piezoelectric resonance sensors is too small for the Fano resonance to be clearly observed. This is the reason why the piezoelectric resonance sensors is usually operated according to the Lorentz spectrum.

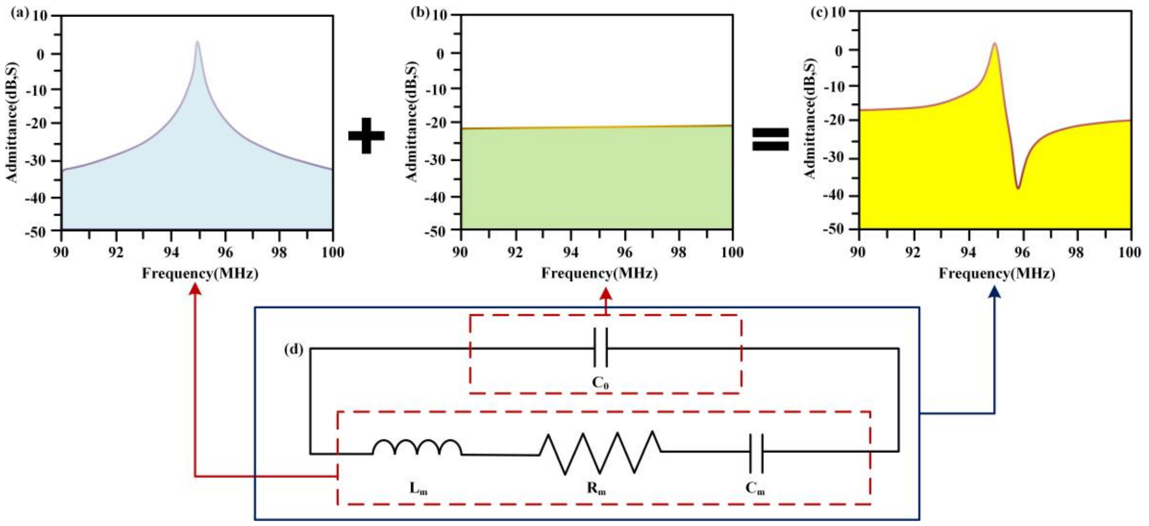

Fig.S1 | (a), the admittance of a series branch. **(b)**, the admittance of a parallel branch. **(c)**, the admittance of SAW resonators. **(d)**, the BVD equivalent circuit for the piezoelectric resonator.

A.2. The admittance of piezoelectric resonance sensors

For a piezoelectric resonator, the admittance is given by

$$Y_{in} = i\omega C_0 + \frac{1}{\frac{1}{i\omega C_m} + i\omega L_m + R_m} \tag{S1}$$

By connecting an external shunt capacitance C_P to the resonator, the static capacitance C_0 is changed to $C_x=C_0+C_P$. The admittance is then written as

$$Y_{in} = i\omega C_x + \frac{1}{\frac{1}{i\omega C_m} + i\omega L_m + R_m} = i\frac{\gamma\lambda}{R_m}\left(\frac{1}{\alpha} + \frac{1}{1-\lambda^2+i\gamma\lambda}\right) \tag{S2}$$

where we define the normalization parameters given by

$$\begin{cases} \lambda = \frac{\omega}{\omega_0} = \frac{2\pi f}{2\pi f_0} = \frac{f}{f_0} \\ \gamma = R_m\sqrt{\frac{C_m}{L_m}} \\ \alpha = \frac{C_m}{C_x} \end{cases} \tag{S3}$$

and

$$\begin{cases} f_0 = \dfrac{1}{2\pi\sqrt{L_m C_m}} \\ Q_m = \dfrac{1}{\gamma} \end{cases}. \tag{S4}$$

In (S4), f_0 is the fundamental resonant frequency of the resonator, and Q_m stands for the quality factor of the series branch.
Without the loss of generality, within the frequency range of interest, f is usually around f_0. Furthermore, $Q_m \gg 1$, leading to $\gamma \ll 1$. We take the approximations (i.e., ignoring high-order terms): $1-\lambda^2 \approx 2(1-\lambda)$ and $\lambda\gamma \approx \gamma$. Eq. (S2) is rewritten as

$$Y_{in} = i\frac{\gamma}{R_m}\left(\frac{1}{\alpha} + \frac{1}{2(1-\lambda)+i\gamma}\right) \tag{S5}$$

The amplitude of the admittance is given by

$$|Y_{in}| = \frac{\gamma}{R\alpha}\sqrt{\frac{\gamma^2 + (2+\alpha-2\lambda)^2}{\gamma^2 + 4(-1+\lambda)^2}} \tag{S6}$$

Letting

$$\frac{\partial |Y_{in}|}{\partial \lambda} = 0, \tag{S7}$$

we yield the series resonant frequency f_s at which the admittance gets the maximum and the parallel resonant frequency f_p at which the admittance gets the minimum,

$$\begin{cases} \lambda_s = \dfrac{1}{4}(4+\alpha-\sqrt{4\gamma^2+\alpha^2}) \approx 1 - \dfrac{\gamma^2}{2\alpha} \\ f_s = \left(1-\dfrac{\gamma^2}{2\alpha}\right) f_0 \approx f_0 \\ \lambda_p = \dfrac{1}{4}(4+\alpha+\sqrt{4\gamma^2+\alpha^2}) \approx 1 + \dfrac{\alpha}{2} + \dfrac{\gamma^2}{2\alpha} \\ f_p = \left(1+\dfrac{\alpha}{2}+\dfrac{\gamma^2}{2\alpha}\right) f_0 \approx \left(1+\dfrac{\alpha}{2}\right) f_0 \end{cases}. \tag{S8}$$

If without C_P, realizing that $\alpha = \frac{C_m}{C_0} \ll 1$, $\sqrt{\frac{C_0+C_m}{C_0}} \approx 1 + \frac{1}{2}\frac{C_m}{C_0}$, Eq.(S8) is then reduced to Eqs. (2) and (3) in the main text.

A.3. The scattering parameter S_{11} of piezoelectric resonance sensors

$$S_{11} = \frac{Y_0 - Y_{in}}{Y_0 + Y_{in}} \tag{S9}$$

where $Y_0 = 1/Z_0$ and Z_0 is the characteristic impedance of the signal source (usually $Z_0 = 50\ \Omega$).
Inserting Eq. (S5) into (S9), it yields the amplitude of

$$|S_{11}| = \sqrt{\frac{\gamma^4 + \gamma^2((-1+R\mathrm{Y}0)^2\alpha^2 - 4\alpha(-1+\lambda) + 4(-1+\lambda)^2) + 4R^2\mathrm{Y}0^2\alpha^2(-1+\lambda)^2}{\gamma^4 + \gamma^2((\alpha+R\mathrm{Y}0\alpha)^2 - 4\alpha(-1+\lambda) + 4(-1+\lambda)^2) + 4R^2\mathrm{Y}0^2\alpha^2(-1+\lambda)^2}} \tag{S10}$$

Letting

$$\frac{\partial |S_{11}|}{\partial \lambda} = 0, \tag{S11}$$

we yield the resonant frequency f_{min} at which $|S_{11}|$ gets the minimum,

$$\begin{cases} \lambda_{min} \approx 1 + \dfrac{\alpha}{2} \\ f_{min} \approx \left[1 + \dfrac{C_m}{2(C_0 + C_p)}\right] f_0 \end{cases}. \tag{S12}$$

A -3 dB bandwidth method of characterizing the quality factor (Q) is used here. It is written as

$$Q \approx \frac{f_{min}}{\Delta f_{3dB}} \quad (S13)$$

where $\Delta f_{3dB} = f_2 - f_1$ is the frequency bandwidth, and f_1 and f_2 are the frequencies corresponding to a response value of $|S_{11}|/|S_{11}|_{min} = \sqrt{2}$. The scattering parameter S_{11} is measured in different external shunt capacitance C_p, and Q is then estimated by Eq. (S13).

B. Simulation of the admittance, scattering parameter, and quality factor of piezoelectric resonance sensors

We perform simulations using ADS (Advanced Design System) software. Parameters for the simulations are extracted from the as-fabricated resonator for humidity sensing. They are, $C_0 = 96.16$ pF, $C_m = 1.737$ pF, $L_m = 1.616$ μH, and $R_m = 2.994$ Ω, respectively. The admittance and scattering parameter have been simulated for different shunt capacitances, and the quality factor is estimated according to Eq. (S13).

Fig. S2 shows the schematic diagram of ADS simulations. Fig. S3 shows the simulated admittance as a function of frequency for different shunt capacitances. Fig. S4 shows the simulated scattering parameter S_{11} as a function of frequency for different shunt capacitances.

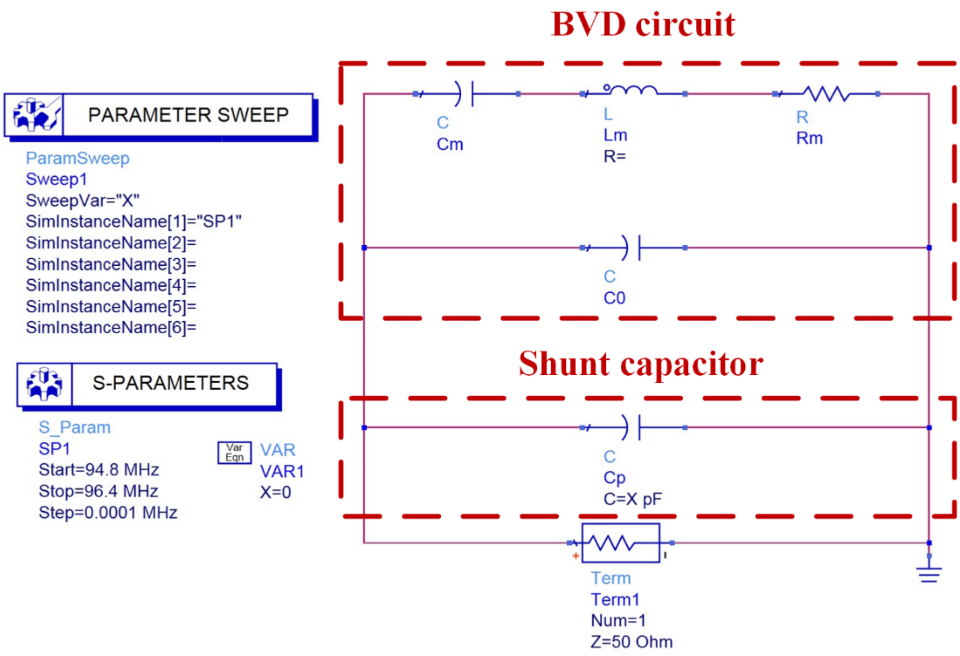

Fig.S2 | The schematic diagram of ADS simulations.

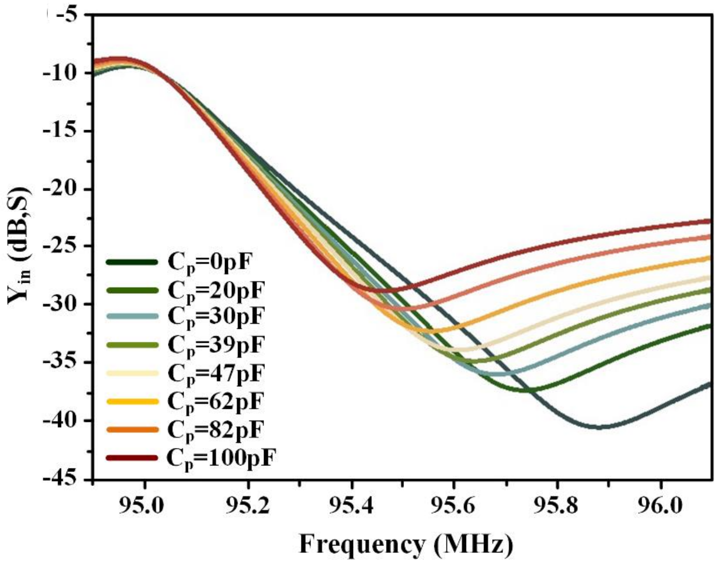

Fig.S3 | The simulated admittance as a function of frequency for different shunt capacitances.

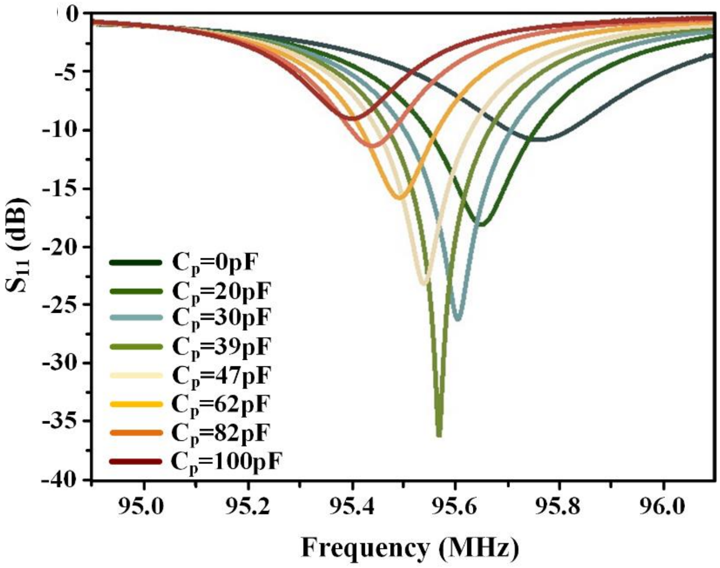

Fig.S4 | The simulated scattering parameter S_{11} as a function of frequency for different shunt capacitances.

From Fig.S3 we have obtained the series resonant frequency and parallel resonant frequency as a function of shunt capacitance, as shown in Fig.S5. It indicates that the results of the ADS simulation are generally in good agreement with the corresponding results obtained experimentally.

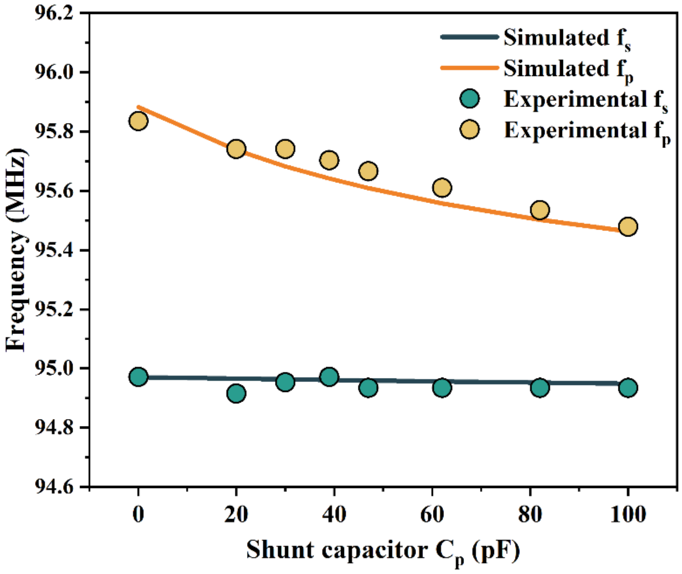

Fig.S5 | The simulated series resonant frequency and parallel resonant frequency as a function of shunt capacitance. Experimental results are also given for comparisons.

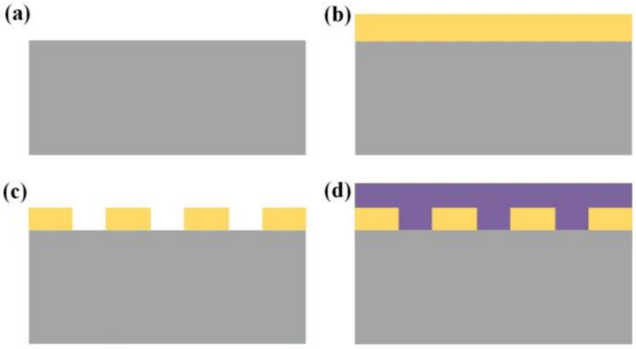

Fig. S6 | The fabrication process of a SAW resonator for humidity sensing, (a) Cleaning of the substrate, (b) Metal deposition, (c) Patterning and etching of structures, (d) deposition of sensitive material.

C. Fabrication process of piezoelectric resonance sensors

The fabricated process of the SAW resonator for humidity sensing is displayed in Fig. S6. The fabrication process started with a 128° Y-X $LiNbO_3$ substrate. An Au film with a Cr adhesion layer was deposited onto the $LiNbO_3$ substrate by an electron beam evaporation process. Then it was patterned and etched to form the SAW structure. Finally, the sensitive material was electrospun on the structure from a DMF (N,N-Dimethylformamide, ACS Spectral grade, ⩾ 99.8%) solution of 20% wt PMMA and 1% wt GO.

D. Experimental set-up

The schematic and photo picture of our test system are shown in Fig.S7.

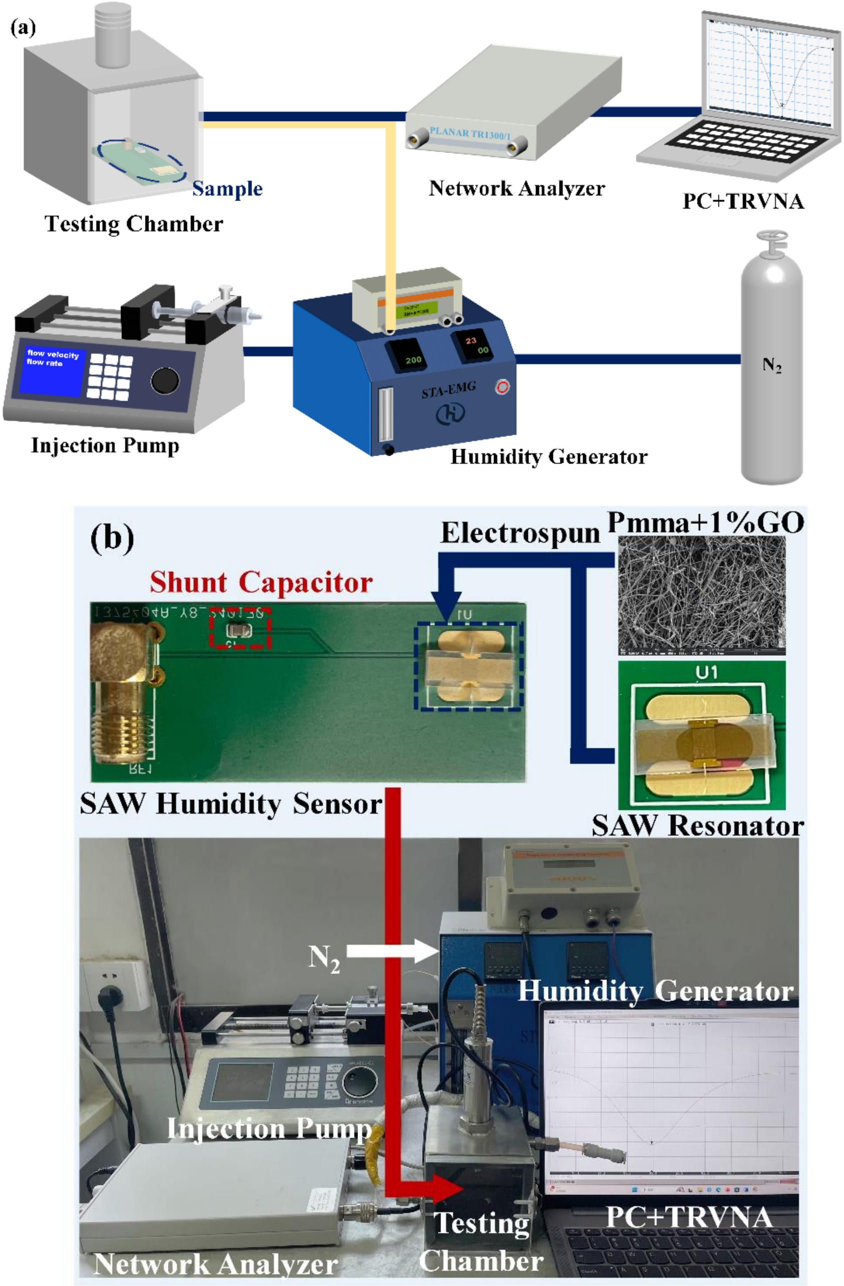

Fig. S7 | Experimental set-up. (a) The schematic of test systems. (b) Photo picture of test systems. Partial view of the SAW sensor covered with a mixture of 20% wt PMMA and 1% wt GO, shunt capacitor, and connections.

The SAW sensor was placed inside a sealed container, which was connected to a humidity generator (Zonho-tech STA-EMG). The humidity generator adjusted humidity levels by varying the ratio of injected water and nitrogen, and a split temperature and humidity transmitter (Race M&C-Tech HTDT2-IESX102) were used for calibration. A vector network analyzer VNA (Copper Mountain PLANAR TR1300/1) was utilized to characterize the output of the sensor. For each shunt capacitor, the test frequency was swept from 94.6 to 96.1 MHz with 1600 sample points. Both adsorption and desorption processes for humidity sensing were measured.
